# Noisy Splicing Drives mRNA Isoform Diversity in Human Cells

**DOI:** 10.1371/journal.pgen.1001236

**Published:** 2010-12-09

**Authors:** Joseph K. Pickrell, Athma A. Pai, Yoav Gilad, Jonathan K. Pritchard

**Affiliations:** 1 Department of Human Genetics, The University of Chicago, Chicago, Illinois, United States of America; 2 Howard Hughes Medical Institute, The University of Chicago, Chicago, Illinois, United States of America; University of Geneva Medical School, Switzerland

## Abstract

While the majority of multiexonic human genes show some evidence of alternative splicing, it is unclear what fraction of observed splice forms is functionally relevant. In this study, we examine the extent of alternative splicing in human cells using deep RNA sequencing and *de novo* identification of splice junctions. We demonstrate the existence of a large class of low abundance isoforms, encompassing approximately 150,000 previously unannotated splice junctions in our data. Newly-identified splice sites show little evidence of evolutionary conservation, suggesting that the majority are due to erroneous splice site choice. We show that sequence motifs involved in the recognition of exons are enriched in the vicinity of unconserved splice sites. We estimate that the average intron has a splicing error rate of approximately 0.7% and show that introns in highly expressed genes are spliced more accurately, likely due to their shorter length. These results implicate noisy splicing as an important property of genome evolution.

## Author Summary

Most human genes are split into pieces, such that the protein-coding parts (exons) are separated in the genome by large tracts of non-coding DNA (introns) that must be transcribed and spliced out to create a functional transcript. Variation in splicing reactions can create multiple transcripts from the same gene, yet the function for many of these alternative transcripts is unknown. In this study, we show that many of these transcripts are due to splicing errors which are not preserved over evolutionary time. We estimate that the error rate in the splicing of an intron is about 0.7% and demonstrate that there are two major types of splicing error: errors in the recognition of exons and errors in the precise choice of splice site. These results raise the possibility that variation in levels of alternative splicing across species may in part be to variation in splicing error rate.

## Introduction

Most mammalian mRNAs are processed from much longer precursors in a series of splicing reactions. Regulation of these splicing reactions can lead to alternatively spliced forms of mRNA from the same pre-mRNA [1], and there is considerable interest in cataloguing the functionally important transcripts of all mammalian genes. Towards this end, transcript diversity has been examined using data from full mRNA sequences, expressed sequence tags (ESTs), or high-throughput sequencing of cDNA libraries (RNA-Seq) [2]–[6]. In particular, recent RNA-Seq studies have established that nearly all multiexonic human genes have multiple detectable isoforms [2], [5].

The observation of extensive alternative splicing could indicate that most genes have many functionally-relevant isoforms; alternatively, many transcripts could be nonfunctional “noise” [7]–[10]. The latter explanation is supported by a few pieces of evidence from analyses of EST databases. In particular, a large fraction of exon-skipping events in human genes is not observed in mice (ie. is not conserved) [11]–[13], and the number of observed isoforms of a gene correlates with the number of exons it has (and thus the theoretical number of potential transcripts it could produce) [8]. Additionally, it is hypothesized that short introns in humans (as well as in other eukaryotes) have evolved to preferentially trigger degradation via nonsense-mediated decay (NMD) mechanisms when the spliceosome fails to remove them, suggesting that such errors are common enough to exert a detectable selective pressure [14].

There are also theoretical reasons to expect splicing to be error-prone. First, the binding sites for proteins important in exon recognition comprise a large mutational target; it has been estimated that approximately 30 bases are necessary for fully efficient splicing of an intron [15]. The potential for mutational disruption of these binding sites has been referred to as part of the “intrinsic cost of introns” [16]. Further, the large size of introns relative to exons gives ample opportunity for the mutational creation of *new* (weak) binding sites. Although mutations which create or disrupt binding sites may be slightly deleterious, the large number of possible such mutations makes it inevitable that some will reach fixation in a population. This is particularly relevant in species, such as humans, with relatively small long-term effective population sizes. It is plausible, then, that the human genome carries a substantial load of suboptimal sequences which cause the generation of aberrant transcript isoforms. In this study, we present direct evidence that this is indeed the case.

## Results

We have performed deep sequencing of cDNA libraries generated from mRNA from 75 lymphoblastoid cell lines derived from Nigerian individuals as part of the International HapMap Project (69 from Pickrell et al. [17] and 6 additional ones). In total, we generated 1.4 billion sequencing reads of either 35 or 46 base pairs. 1.2 billion of these sequencing reads mapped to the genome. We used the remainder to identify splice junctions (without reference to known exons) by splitting each sequencing read into two and mapping each end to the genome independently. In total, 48 million additional reads mapped to the genome using this read-splitting procedure, and we identified 392,612 putative splice junctions.

**Figure 1:**
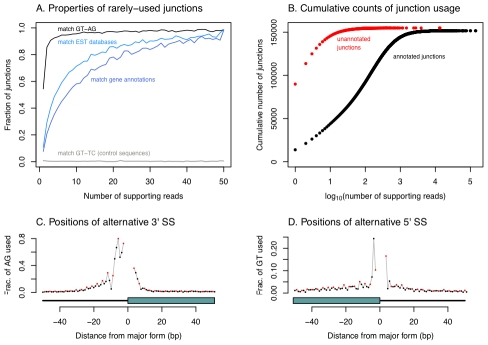
Extensive unannotated splicing in human cells. A. We plot, as a function of number of supporting reads, the fraction of junctions 1) matching GT-AG, the splice site consensus sequences (black), 2) matching a control pair of dinucleotides (grey), 3) annotated in EST databases (light blue), or 4) annotated in gene databases (dark blue). B. We split all junctions into those that are annotated in gene model databases and those that are not. Plotted is the cumulative number of junctions of each type by expression level. Unannotated junctions are expressed at much lower levels than annotated junctions. C and D. Alternative splice junctions near known protein-coding junctions show a periodic pattern. At each alternatively-spliced protein-coding 3′ or 5′ splice site, we counted the positions of AG (or GT, respectively) dinucleotides used as alternative splice sites, then averaged this across splice sites (see Methods). The red points denote positions that are a multiple of three base pairs from the major splice form, and the black points those that are not. The blue box below each panel shows the position of the exon.

Most previous investigations of splice junctions using RNA-seq data have considered putative splice junctions only between previously annotated or predicted exons [2], [4], [5], [18], [19]. Since our method makes no such restriction, one reasonable concern is that we might identify spurious junctions due to mapping or sequencing errors. However, there is strong evidence that the identified junctions are real outcomes of splicing reactions. First, despite the fact that our mapping approach used no information about the sequence specificity of the splicing reaction, the majority of the putative junctions contain GT-AG dinucleotides (the canonical splice sites) directly intronic of the edges of the predicted splice sites (Figure 1A). If we assume that all putative junctions without intronic matches to the canonical splice sites (or the alternative dinucleotide pair GC-AG) are false positives, we estimate a False Discovery Rate (FDR) of 1.5% for the 306,606 junctions that do contain such matches (Methods). Second, the positions of putative alternative splice sites near protein-coding splice sites follow a periodic pattern, such that splice sites which maintain the coding frame of the exon are observed more often than those which disrupt frame (Figure 1C, 1D). In total, 42% of these alternative splice sites are in frame (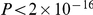; binomial test against the null hypothesis of 33%). This observation recapitulates patterns seen in studies of EST databases [20]–[22]. In the remainder of the paper we analyze only the set of 306,606 junctions which contain intronic matches to GT-AG or GC-AG.

### Many identified junctions are novel

**Figure 2:**
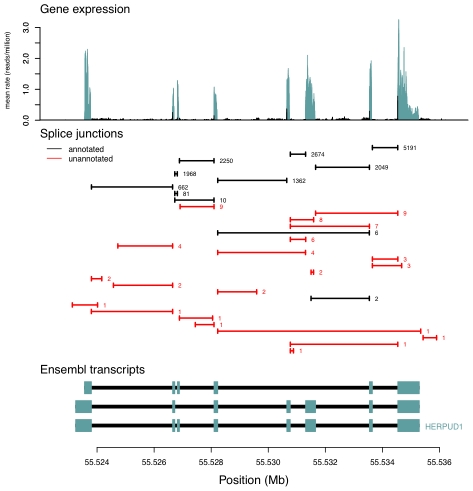
An example of splice junctions identified in a gene. In the top panel, we plot the average expression level at each base in a region surrounding *HERPUD1*. In blue are bases annotated as exonic, and in black are those annotated as not exonic. In the middle panel, we plot the positions of all splice junctions in the region identified in our data. In black are splice junctions that are present in gene databases; in red are those that are not. The number of sequencing reads supporting each junction is written to the right of each junction, and junctions are ordered from top to bottom of the plot according to their coverage. In the bottom panel, we show the gene models in the region from Ensembl. The blue boxes show the positions of exons, and the black lines the positions of introns.

We compared the junctions we identified to gene models from the UCSC, Ensembl, Vega, and RefSeq databases, and to spliced ESTs from Genbank [23]–[27]. Of the 306,606 splice junctions, 154,927 (50.5%) are not annotated as parts of known gene models, and 136,313 (44.5%) are not present in Genbank. For splice junctions not present in gene models, we estimate an FDR of about 2% (Methods). The extensive unannotated splicing we observe is largely due to junctions that are rarely seen in our data (Figure 1B): while 50% of all observed junctions are not present in gene models, these account for only 1.7% of all junction-spanning sequencing reads (Table 1). For example, 21 of the 32 splice junctions observed in the gene *HERPUD1* are unannotated, but only around 0.5% of the reads from this gene are derived from these 21 unannotated junctions (Figure 2). We see no sign that our identification of isoforms is near saturation (Figure S1); thus deeper sequencing of transcriptomes will likely continue to identify additional low-abundance isoforms.

**Table 1:** Characteristics of observed junctions.

	Both ends known				
	Known junc.	New junc.	New 3′ SS	New 5′ SS	Both new
number	151,679	27,611	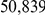	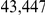	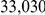
mean coverage	255	5	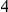	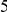	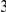
% obs. in other tissues	87	27	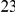	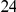	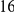
% near known [5′,3′]	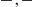	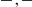	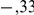	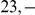	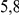
% highly conserved [5′,3′]	76, 76	75, 77	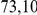	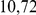	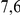

As described in the main text, we split the observed junctions into five classes based on gene model databases. For each class, we present the number of such junctions, the average number of reads spanning each junction in that class, the percentage of the junctions observed in any tissue assayed in Wang et al. [2], the percentage of 5′ and 3′ splice sites of each junction that fall near an annotated splice site (“near” here is defined as within 50 base pairs), and the percentage of the 5′ and 3′ splice sites of each junction that show strong evidence of evolutionary conservation (defined as a mean *phyloP* score 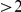 [31] at the two canonical bases of the splice site).

### Characteristics of identified junctions and splice sites

Next, we quantified overall levels of alternative splicing. To do this, we considered a set of splice sites covered by at least 50 reads in our data (there are 77,754 such 5′ splice sites and 77,733 such 3′ splice sites; 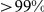 of these are annotated in gene model databases). We then counted both the number of different places in the genome to which each site is spliced, as well as the proportions of reads covering each junction. We estimate that the “major” splice form accounts for 98.4% of reactions involving each splice site; in our data the average splice site is involved in 1.8 different splicing reactions. 5′ splice sites in untranslated regions (UTRs) are involved in a mean of 2.6 splicing reactions, versus 1.8 for 5′ splice sites in protein-coding regions; the corresponding numbers are 3.2 and 1.7 for 3′ splice sites.

We then evaluated how the splice junctions correspond to known gene models. We split the junctions into five classes: (i) those where the junction is annotated; (ii) those between splice sites that are both annotated (but not annotated as being spliced together); (iii) those where the 5′ splice site (but not the 3′ splice site) is annotated; (iv) those where the 3′ splice site (but not the 5′ splice site) is annotated; and (v) those where neither splice site is annotated. 80% of the unannotated junctions involve at least one annotated splice site, and though many newly-identified splice sites fall near annotated sites, the majority do not (Table 1). This indicates that a large fraction of the low-abundance isoforms are not modifications of known exons, but instead contain entirely new exons.

### Extensive unannotated splicing is present in different human populations and tissues

We next asked whether we could confirm these observations in other cell lines and primary tissues. We first performed the same analysis on RNA-Seq data generated on a different set of human LCLs [28]. We identified 219,322 splice junctions at an FDR of 1.5%, 82,658 of which are unannotated (Figure S1). We then analyzed RNA-Seq data from primary human liver samples (George Perry, unpublished data); we identified 156,905 splice junctions at an FDR of 0.8%, 29,655 of which are unannotated (Figure S1). Finally, we analyzed RNA-Seq data from a set of several primary tissues [2]; we identified 136,499 splice junctions at an FDR of 1.8%, 21,743 of which are unannotated (Figure S1). The numbers of unannotated splice junctions in these studies are roughly consistent with the observed numbers found in the Yoruba data, given the lower sequencing depths of those other studies and hence better sampling of common junctions relative to rare junctions (Figure S1). This confirms that the observation of extensive unannotated splicing is broadly generalizable; for the rest of the paper we focus on the original set of Yoruban LCL data since this is the largest RNA-Seq dataset currently available.

We also considered how much splicing shows evidence of being restricted to particular individuals, rather than shared across the entire population (due to, for example, sequence polymorphisms which influence splicing [17], [28]–[30]). Though it is difficult to estimate this precisely, several analyses suggest that only a couple percent of splice junctions, at maximum, show evidence of being restricted to certain individuals (Text S1).

### Most unannotated splice junctions show no evolutionary conservation

We hypothesized that unannotated, rarely-used splice sites are the result of evolutionarily-neutral (or perhaps slightly deleterious) splicing errors. To test this, we compared the sequence conservation across placental mammals (using the *phyloP* score [31]) between the unannotated and annotated splice sites (we assume that current gene databases are highly enriched for truly functional exons). If the unannotated splice sites are functionally relevant, their sequence conservation should be comparable to that of annotated splice sites. For this analysis, we used the set of splice junctions where one end is an annotated splice site and the other is more than 50 bases away from an annotated splice site.

**Figure 3:**
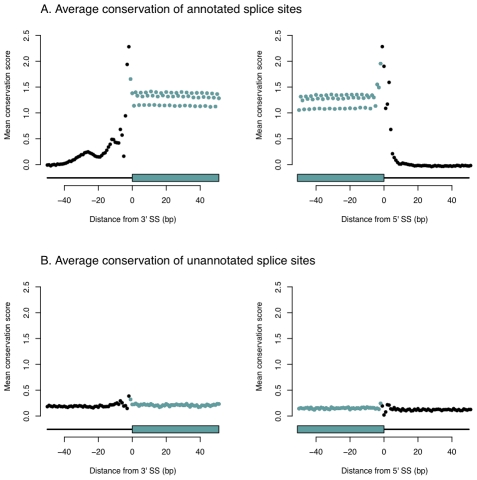
Unannotated splice junctions show little evidence of evolutionary conservation. In each panel, we plot the mean *phyloP* score [31] at each base surrounding the splice site. In the top panels are annotated splice sites, and in the bottom panels are unannotated splice sites. In blue are bases exonic of the splice site, and in black are those intronic of the splice site, as diagrammed below each panel.

Annotated splice sites show a striking pattern of sequence conservation–the splice sites themselves are highly conserved, as are the regions exonic of the splice sites (Figure 3A). In contrast, the conservation scores around unannotated splice sites show little, if any, signal of evolutionary constraint (Figure 3B). This same pattern holds in an independent sample of LCLs and in primary human liver samples (Figures S2 and S3). To exclude the possibility that our analysis overlooked splice sites that are conserved in only a subset of placental mammals, we repeated this analysis using *phyloP* scores calculated using only primates, and saw the same pattern (Figure S4). Further, humans have reduced polymorphism in the annotated splice sites, but no such reduction in the unannotated splice sites (Figure S5). The most parsimonious explanation of these observations is that the majority of rarely-used, unannotated splice sites are simply due to mis-spliced transcripts.

### Estimation of splicing error rates

To estimate the fraction of mature mRNAs resulting from mis-splicing, we identified a set of splice junctions where both the 5′ and 3′ splice sites are highly conserved (those with a *phyloP* score 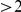). We then asked how often the conserved splice sites are spliced to unconserved splice sites (to provide a conservative lower bound on the amount of mis-splicing, we used a relaxed threshold of a *phyloP* score 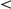 0.5 to call a splice site as unconserved for this analysis). Approximately 0.7% of reads involving either end of a conserved junction are to unconserved splice sites. Given that the median gene in the human genome has four exons (and thus three splicing reactions), this suggests that approximately 2% of transcripts from the average gene are mis-spliced. Because mis-spliced transcripts are preferentially removed by NMD mechanisms, this is likely a conservative estimate.

**Figure 4:**
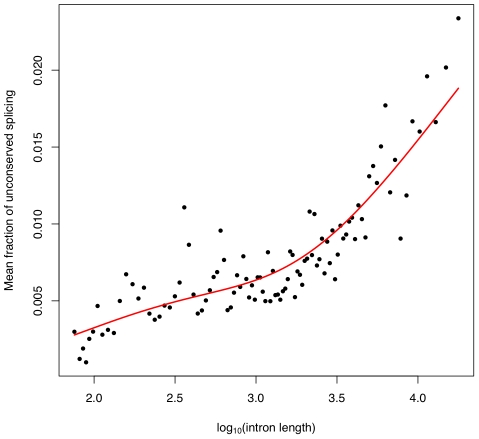
Splicing error rate correlates with intron length. We divided all introns that are bounded by highly conserved splice sites into 100 bins based on length. We then calculated, in each bin, the mean fraction of sequencing reads from either splice site to an unconserved splice site. Plotted is this mean against the 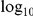 of the mean intron length (in base pairs) of introns in the bin. In red is a spline fit to these points.

We next tried to identify factors that are predictive of an intron's level of splicing error. The two factors we considered were the intron's length and the expression level of the gene in which the intron falls. Longer introns show higher levels of mis-splicing (Figure 4), while highly expressed genes show somewhat lower levels (Figure S6). These associations may be confounded, however, by the fact that highly expressed genes tend to have shorter introns [32]. Indeed, the association between splicing error rate and gene expression level disappears after correction for intron length (Figure S5), indicating that this association is largely driven by the lower splicing error rate of small introns. The different sequence composition of introns in highly expressed genes [33] may also influence their lower rate of splicing error (Text S1; Figure S7).

### “Noise” splice sites are marked by genomic features that define exons

**Figure 5:**
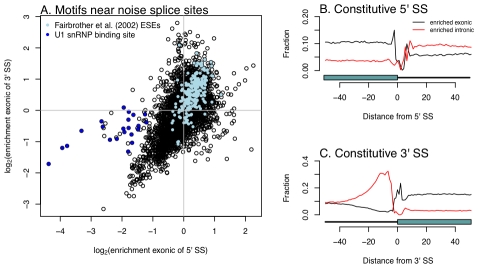
Hexamers enriched near unconserved splice sites are relevant in exon definition. A. Plotted is the 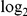 enrichment of all possible hexamers exonic of either 5′ or 3′ noise splice sites. In light blue are hexamers identified as exonic splicing enhancers by Fairbrother et al. [35], and in dark blue are hexamers that are good matches to the consensus U1 snSNP binding site (we include all hexamers matching five contiguous bases of “AGGTAAG”). B and C. Hexamers from A. mark borders of constitutively spliced exons. Each point is the fraction of hexamers starting at that position relative to a constitutively spliced exon (in these cells) which match the hexamers identified as significantly enriched exonic or intronic of the “noise” 5′ or 3′ splice sites.

Finally, we considered the mechanism which results in the generation of mis-spliced transcripts. To do so, we looked for hexamers enriched in the vicinity of unconserved, rarely-used splice sites, as compared to nearby “decoy” splice sites (matching GT or AG) which we never observed to be used in our data. 574 hexamers show significant enrichment or depletion exonic of the 5′ SS, and 728 exonic of the 3′ splice site (Figure 5A; 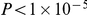; 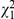 test). Although the relative enrichments of hexamers near the 5′ and 3′ splice sites are similar, a set of hexamers matching the binding site for the U1 snRNP (which recognizes the 5′ splice site) is strongly depleted in the vicinity of the 5′ splice sites, but shows only limited or no depletion in the vicinity of 3′ splice sites (Figure 5A). This is consistent with the observation that competitive binding of the core splice factors plays an important role in splice site choice [34]. There is a smaller, but still substantial, number of hexamers enriched or depleted intronic of the splice sites (295 and 282 for 5′ and 3′ splice sites, respectively).

We compared our list of hexamers to the list of exonic splicing enhancers (ESEs) identified by Fairbrother et al. [35], some of which were validated experimentally, and found that 79% of the ESEs are enriched exonic of both 3′ and 5′ splice sites in our data (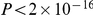, 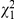 test). This is evidence that noisy splicing is a result of the binding of the same splice factors used to identify exons more generally. Further support for this possibility comes from the observation that the hexamers identified near noise splice sites demarcate the boundaries of constitutive exons in our data (Figure 5B, 5C).

## Discussion

In this study, we have examined the extent of alternative splicing in human cells. We have demonstrated that there is a large number of evolutionarily unconserved (and presumably slightly deleterious) alternative isoforms of most genes, and have presented evidence that this noisy splicing is the result of stochastic binding of sequence-specific factors involved in exon recognition.

### Noisy splicing

Two main observations support the contention that the majority of low-abundance isoforms are due to splicing errors. First, rarely-used splice sites are enriched near often-used splice sites, and additionally show a periodic pattern around those sites ([20], [21], Figure 1). Our interpretation of these observations (like that of Dou et al. [20] and Chern et al. [21]) is that the splicing machinery occasionally misses its “intended” splice site, and that the resultant isoforms which disrupt the protein-coding reading frame are preferentially degraded by the NMD machinery. Second, rarely-used, unannotated splice sites (we interpret the fact that a splice site is annotated as evidence that it is relatively highly expressed in at least one tissue) show little evidence of evolutionary conservation across placental mammals (Figure 3) or primates (Figure S3), or of constraint within humans (Figure S4). We conclude, then, that the majority of low-abundance splice forms are indeed “noise”. In fact, because our identification of splice forms is not yet at saturation (Figure S1), we extrapolate that the majority of different mRNA isoforms present in a cell are not functionally relevant, though most copies of a pre-mRNA produce truly functional isoforms. We speculate that this conclusion will hold at the protein level as well.

### Mechanisms of noisy splicing

One goal in understanding the mechanism of splicing is the generation of a “splicing code”–a set of rules which the cell uses to convert the information present in a pre-mRNA sequence to a properly spliced mRNA [36]–[38]. This code presumably involves the binding sites for various splice factors [35], [39], [40], as well as local chromatin structure [41]–[46]. The results presented here suggest that, instead of being a deterministic function that maps a pre-mRNA sequence to a spliced transcript, the “splicing code” instead defines a probability distribution for each pre-mRNA on a large number of possible splice forms. That is, the same pre-mRNA sequence will stochastically result in a large number of isoforms, presumably even within the same cell. We suggest that low-probability events may be informative about the parameters of this distribution.

### Evolutionary consequences of noisy splicing

The level of splicing error observed in a gene reflects a balance between the continuous input of mutations that disrupt splicing and the ability of selection to remove them [16]. Indeed, selection for proper discrimination between introns and exons has affected genome evolution in a number of ways, by constraining the composition of amino acids coded near splice sites [47] and influencing the sequence composition of introns [14], [48]. We have shown that longer introns are more prone to splicing errors. This is consistent with the increased rate of birth of new, alternatively spliced exons in long introns [49], [50], and supports the contention that long introns are more deleterious than short introns [51], [52]. This may contribute to selection for short introns in highly expressed genes [32].

One implication of the above reasoning is that the level of splicing error observed in an organism should depend on the complexity of the splicing machinery in the species (ie., the number of potential mutations that could affect splicing) and the effective population size of the species (and hence the effectiveness of natural selection in removing those mutations). This is consistent with the observation that levels of alternative splicing vary considerably across eukaryotes [53]. A prediction, then, is that species with larger effective population sizes or simpler splicing mechanisms should have lower rates of splicing error. Progress in RNA-Seq technology will soon allow relatively unbiased exploration of the evolution of splicing noise in a wide variety of species, and allow propositions such as these to be tested.

## Methods

### Data used

For the main analysis of LCLs, we used RNA-Seq data generated on 75 HapMap cell lines derived from Yoruban individuals. Data from 69 of these were reported in Pickrell et al. [17]. We also generated RNA-Seq data on six additional HapMap Yoruban cell lines, using the same protocol as in Pickrell et al. [17]. Each cell line was sequenced in two lanes on the Illumina GA2 platform, one lane at the Yale sequencing center using 35 base pair sequencing reads, and one at the Argonne sequencing center using 46 base pair sequencing reads. The cell line identifiers and basic quality control metrics are presented in Table 1 in Text S1. All data are available at http://eqtl.uchicago.edu.

For the analysis of an independent set of human LCLs, we obtained RNA-Seq data on a set of 60 HapMap cell lines derived from individuals of European descent [28]. These data consist of paired-end 37 base pair reads generated on the Illumina GA2 platform, obtained from http://jungle.unige.ch/rnaseq_CEU60/. We treated each end of a paired-end read independently, and used the protocol described below to identify splice junctions. For the analysis of primary human liver cell, we used RNA-Seq data from four human liver samples (G. Perry, unpublished). These data consist of paired-end 76 base pair reads generated on the Illumina GA2 platform. RNA-Seq data from multiple other tissues were obtained from Wang et al. [2] and Wu et al. [54]. All data were processed in the same manner, described below.

### Identification of splice junctions *de novo*

Here, we describe our approach for *de novo* identification of splice junctions, which is similar to previous approaches [17], . Software is available at http://eqtl.uchicago.edu.

1. First, we mapped all the reads to a modified version of hg18, where all chromosomes labeled “random” were removed and the pseudoautosomal region of the Y chromosome was converted to all “N”s. We used bwa v.0.5.7 [61] with the default parameters.
2. All reads that did not map to the genome in the first step were considered as possible junction-spanning reads. We took the first 20 bases and the last 20 bases of each such read and mapped each end to the genome independently using bwa. For reads shorter than 40 bases, these two ends are overlapping.
3. If both ends of a read shorter than 40 bases mapped uniquely to the genome, we discarded the read. If both ends of a longer read mapped uniquely (with a mapping quality score 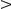
10) to the same strand of the same chromosome within 20 kb (and greater than 50 bases apart), we considered all possible splice junctions consistent with the positions of the two ends and reported all junctions with the lowest number of mismatches between the read and the genome.
4. If one end of a read mapped uniquely to the genome, we first extended the alignment as far as we could without allowing a mismatch, then searched 20 kb downstream for a perfect match to the rest of the read. If there was only one such perfect match, and there were at least 10 bases mapped on either side of the splice junction, we kept the read.
5. We note that there is ambiguity in the precise splice site covered by a read. This is because the consensus 5′ splice site is *AG* 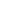 GTA (where 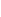 denotes the position of the splice junction, and italics the exonic sequence), and the consensus 3′ splice site is CAG 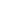 *G*. So, for example, a read covering that splice junction would also be consistent with a 5′ splice site of *A* 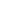 GGTA and a 3′ splice site of CA 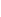 *GG*. For each read which we were able to map, we recorded all such possible splice junctions. In the majority of the paper, however, we use only those junctions matching GT-AG or GC-AG.

By doing the mapping in this way, we expect to recover approximately 55% of all the 46 base pair reads that span splice junctions (outside repetitive regions of the genome), and approximately 30% of all the 35 base pair reads that span splice junctions (there are 10 allowable breakpoints in a 35 base pair read, and 26 allowable breakpoints in a 46 base pair read). We note that we are also limiting ourselves to identifying introns with a maximum length of 20 kb; this is sufficient for the majority of introns in humans.

### Analysis of lowly-used splice sites near protein-coding sites

In Figure 1, we show the density of splice sites near known protein-coding sites. In this analysis, we used only splice sites annotated as being protein-coding in all the transcripts of the gene in Ensembl and Refseq. To generate this figure for the 3′ splice sites, we identified all the 5′ splice sites covered by at least 20 reads, spliced to at least two 3′ splice sites, and where one of those 3′ splice sites contributed 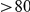
% of all the reads from that 5′ splice site. We will call that 3′ splice site the “major” splice site, and the other the “minor” splice site. We then recorded all the positions of matches to AG in the region surrounding the “major” splice site. For each distance from the “major” splice site, we can then count the number of “minor” splice forms at that distance, as well as the number of AG dinucleotides that would lead to a splice site at that distance. The ratio of those two numbers is plotted in Figure 1. We excluded the positions from −2 to +2 from the splice site due to ambiguity in the read mapping. Analysis for the 5′ splice sites is analogous.

### Genome annotations used

We downloaded the Ensembl, UCSC, Vega, and RefSeq gene models from the annotation of hg18 in the UCSC Genome Browser on Dec. 31st, 2009. We downloaded the spliced EST track on March 1st, 2010. Throughout the paper, when we refer to an “annotated” splice junction, we mean one present in at least one of the Ensembl, UCSC, Vega, or RefSeq databases as of that date. In several places in the paper, we use presence of a splice site in these databases as a proxy for function (rather than, for example, read depth in our data). This is supported by our analysis of conservation (Figure 3A); even rarely-used splice sites in LCLs which are present in gene databases show high levels of sequence conservation (Figure S8). This is likely due to that fact that some fraction of rarely-used splice sites in LCLs are abundantly used in some other tissue, and thus are both functionally relevant and annotated in current databases.

### Estimation of the False Discovery Rate of splice junctions

We can estimate the FDR for the junctions we identified by considering how often each junction is consistent with a GT-AG intron versus control pairs of dinucleotides (recall that each junction read is usually consistent with several pairs of potential splice sites). Of the 392,612 junctions initially identified, 306,606 are consistent with GT-AG or GC-AG (298,346 are consistent with the former, and 8,260 with the latter). In contrast, 4,230 are consistent with control dinucleotides GT-TC or GC-TC (note that the controls simply contain the complement of the 3′ splice site consensus dinucleotide). If we assume that all of the controls are false positives, this gives an FDR of 1.4% (4,230/306,606). If we restrict ourselves only to the 240,644 splice junctions that have not been previously observed, 154,927 are consistent with GT-AG or GC-AG, and 2,985 are consistent with the control pairs of dinucleotides. This gives an FDR for the set of unannotated junctions which contain intronic matches to GT-AG or GC-AG as 1.9% (2,985/154,927). We saw no evidence of enrichment for AT-AC introns, and so did not consider them.

### Conservation analysis

In the analysis of sequence conservation, we used phylop scores generated on the 44-way vertebrate alignment, and downloaded from the UCSC Genome Browser [31]. There are three sets of scores available (representing scores for constraint in all vertebrates, all placental mammals, and all primates); in the analysis presented in the main text, we used scores generated using the placental mammals.

### Analysis of correlates of splicing error rate

For each intron with highly conserved splice sites, we counted the fraction of reads from either splice site to an unconserved splice site, as described in the main text. To estimate the expression level of each gene, we used both our RNA-Seq data and that from the different tissues assayed by Wang et al. [2]. For each tissue, we divided the number of reads mapping to exons of each gene by the length of the exons of the gene. We then took the maximum expression level across tissues as the expression level of the gene for this analysis.

### Motif finding

To look for hexamers enriched around “noise” splice sites, we used a set of splice junctions where one end of the splice junction is to an annotated splice site, and the other is to an unannotated, unconserved (*phyloP* score 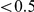) splice site. To be conservative, we removed all the unconserved splice sites within 50 bases of an annotated splice site. Then we identified a set of control sites–for each unconserved splice site, we found an unused GT (or AG) dinucloetide between the used splice site and the nearest annotated one. We then extracted 100 bases around both the used splice sites and the control sites, and counted the frequencies of hexamers in each class (for the 3′ splice site, we excluded the 20 bases intronic and 2 bases exonic of the splice site from this analysis; for the 5′ splice site, we excluded 5 bases intronic and 5 bases exonic). We did this separately for 3′ and 5′ splice sites, and for the intronic and exonic regions of both types of site. Significance was assessed by a 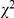 test.

## Supporting Information

Figure S1 Identification of isoforms is not at saturation. We subsampled the junction-spanning reads from the LCL data and asked how many splice junctions we discovered at varying read depths. In black we plot the number of unannotated junctions identified as a function of the number of junction-spanning reads sampled. In other colors are the corresponding numbers for data sets from different tissues.
(0.01 MB PDF)

Figure S2 Unannotated splice sites identified in liver are unconserved. This figure is identical to Figure 3 in the main paper, except that it uses splice sites identified in the liver, rather than the LCL, data.
(0.05 MB PDF)

Figure S3 Unannotated splice sites identified in a different population of LCLs are unconserved. This figure is identical to Figure 3 in the main paper, except that it uses splice sites identified in the European-ancestry LCLs.
(0.05 MB PDF)

Figure S4 Unannotated splice sites are not conserved across primate evolution. This figure is identical to Figure 3 in the main paper, except we used phyloP score calculated in the primate phylogeny, rather than the mammalian phylogeny.
(0.05 MB PDF)

Figure S5 Unannotated splice sites show no reduction of polymorphism levels in humans. We used data from the 1,000 Genomes Project to calculate the SNP density in and around splice sites. For each splice site (annotated or unannotated), we used the SNP calls in the Yoruban population to evaluate whether there is any polymorphism at each position at a distance from each site. Plotted is that fraction of sites that have a polymorphism in the population at each position away from the splice site. Annotated and unannotated splice sites are plotted separately. There is a clear reduction of polymorphism directly intronic of the annotated splice sites, but no such reduction intronic of the unannotated splice sites.
(0.05 MB PDF)

Figure S6 The low splicing error rate of highly-expressed genes is largely due to their small intron sizes. A. Splicing error rate correlates with intron length. This is simply a re-plotted version of Figure 3 in the main text. All highly-conserved introns were grouped into 100 bins based on length; plotted is the mean splicing error rate in the bin against the mean intron length in the bin. B. Correction for gene expression level does not influence the correlation between splicing error rate and intron length. We corrected the observed splicing error rates for gene expression level (see Text S1), and performed the same analysis as in A. C. Splicing error rate correlates with gene expression level. All highly-conserved introns were grouped into 100 bins based on the gene expression level of the gene in which they fall; plotted is the mean splicing error rate in the bin against the mean expression level in the bin. D. Correction for intron length removes the correlation between splicing error rate and expression level. We corrected the observed splicing error rates for intron length (see Text S1), and performed the same analysis as in C.
(0.04 MB PDF)

Figure S7 Sequence analysis of introns. A. 5′ splice site motif density in introns correlates with gene expression level. We calculated the density of matches to the 5′ splice site motif (see Text S1) in each intron, then grouped all introns into 200 bins based on the expression level of the gene in which each falls. Plotted is the mean density of matches to the motif against the mean expression level in each bin. B. 3′ splice site motif density in introns correlates with gene expression level. The same plot as in A., except the y-axis is the density of matches 3′ splice site motif. C. ESE hexamer density in introns correlates with gene expression level. As in A., except the y-axis is the density of matches to the putative ESEs identified by Fairbrother et al. (2002). D. Pseudo-exon density in introns correlates with gene expression level. As in A, except the y-axis is the density of pseudo-exons (see ).
(0.06 MB PDF)

Figure S8 Rarely-used but annotated splice sites are highly conserved. As for Figure 3 in the main text, we identified all the splice junctions where one end is annotated and the other is not. We then limited ourselves to splice sites covered by exactly one read in our data (there are approximately 10,000 such annotated splice sites and 20,000 unannotated splice sites of each type), and performed the same analysis of conservation in the main text. The top panel shows the results for the annotated splice sites, and the bottom panel the results for the unannotated splice sites. The marked difference between the two classes remains.
(0.05 MB PDF)

Text S1 Supplementary text.
(0.09 MB PDF)
