# Supplementary figures and images for: Noisy Splicing Drives mRNA Isoform Diversity in Human Cells
Source: PLoS Genet. 2010 Dec 9;6(12):e1001236. doi: 10.1371/journal.pgen.1001236 (PMC3000347; doi:10.1371/journal.pgen.1001236)

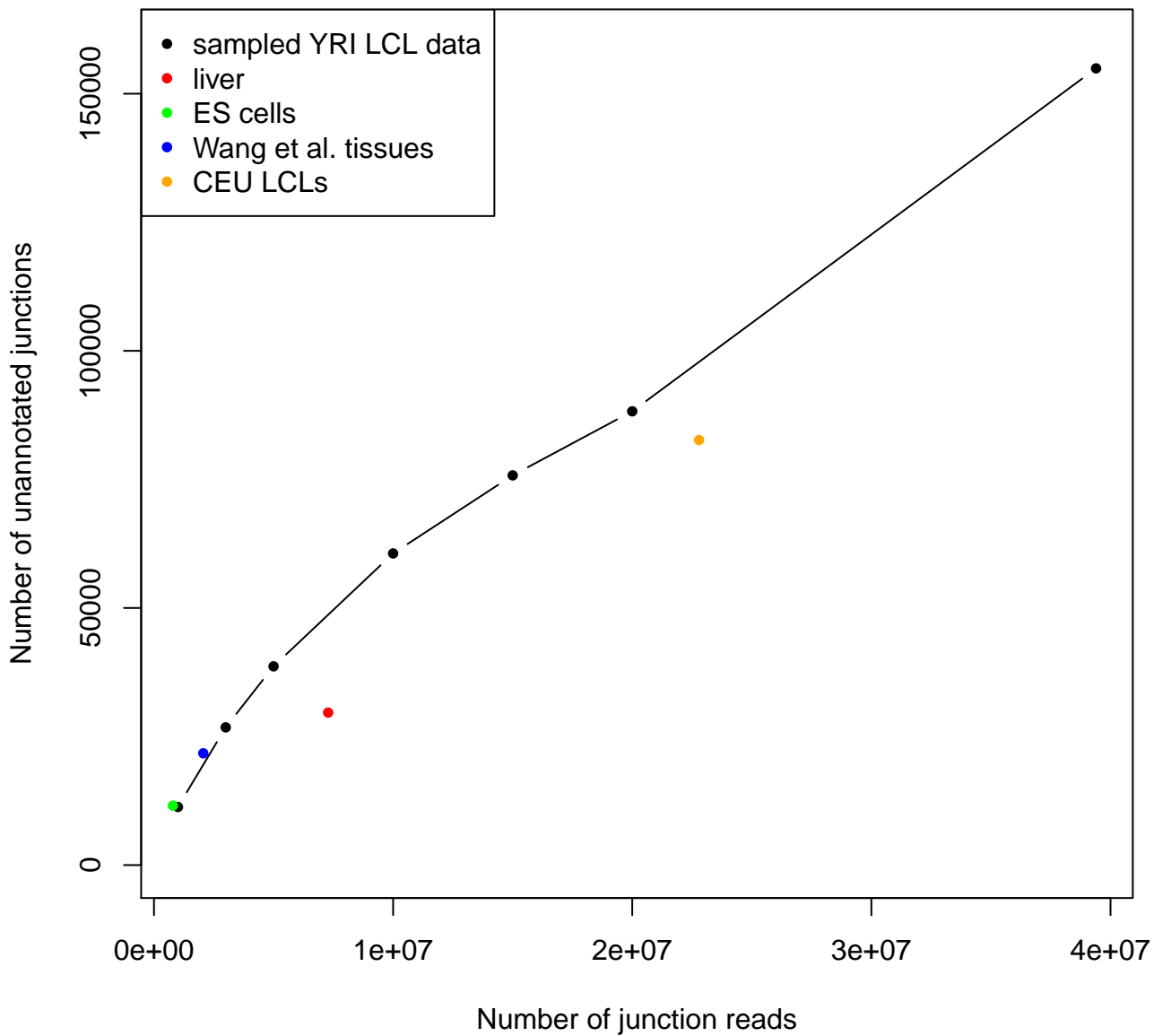

Supplement: Figure S1 — Identification of isoforms is not at saturation. We subsampled the junction-spanning reads from the LCL data and asked how many splice junctions we discovered at varying read depths. In black we plot the number of unannotated junctions identified as a function of the number of junction-spanning reads sampled. In other colors are the corresponding numbers for data sets from different tissues. (0.01 MB PDF) [file pgen.1001236.s001.pdf]

## Annotated splice sites (liver)

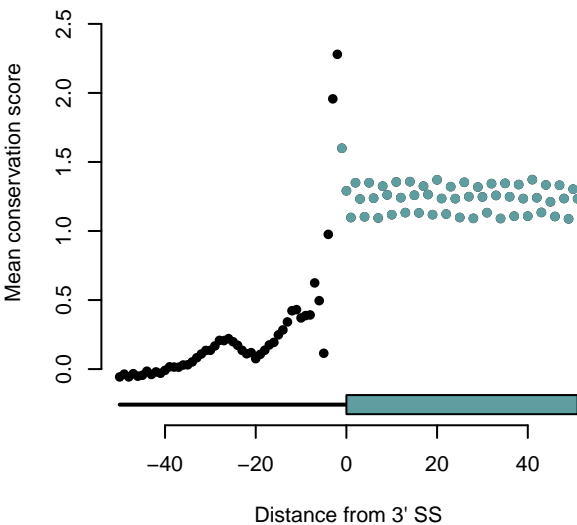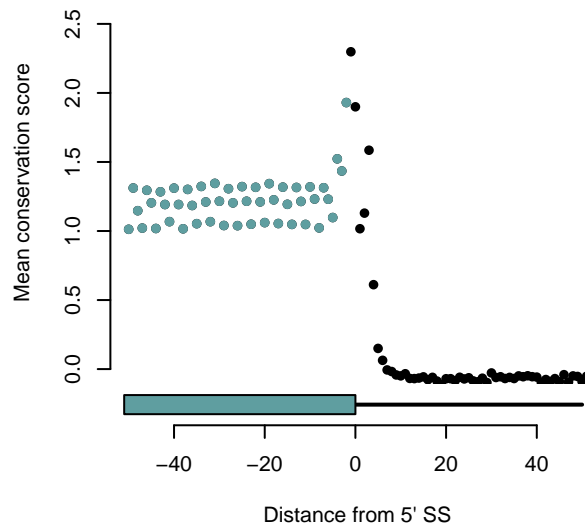

## Unannotated splice sites (liver)

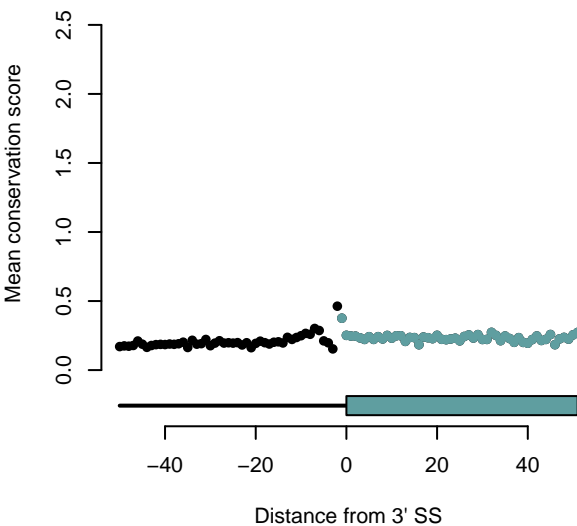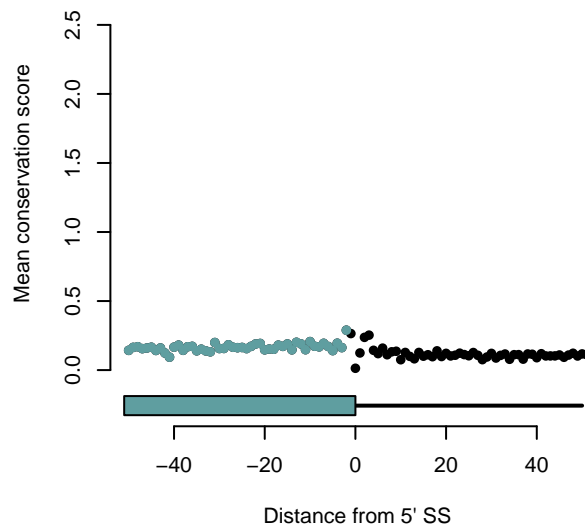

Supplement: Figure S2 — Unannotated splice sites identified in liver are unconserved. This figure is identical to Figure 3 in the main paper, except that it uses splice sites identified in the liver, rather than the LCL, data. (0.05 MB PDF) [file pgen.1001236.s002.pdf]

## Annotated splice sites (CEU LCLs)

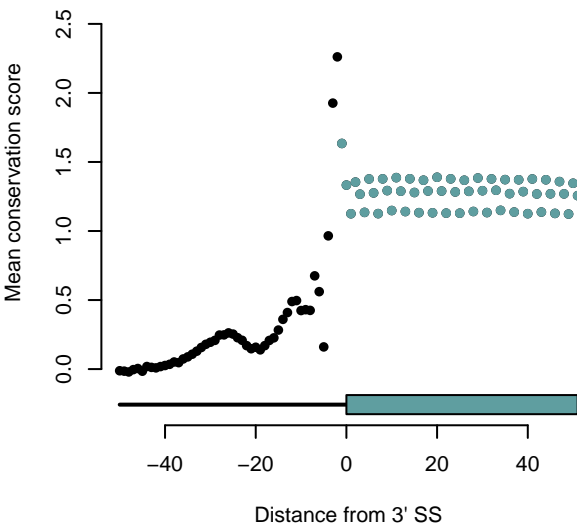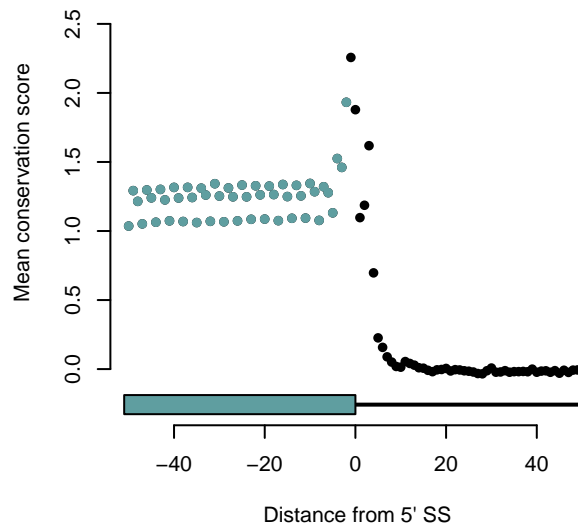

## Unannotated splice sites (CEU LCLs)

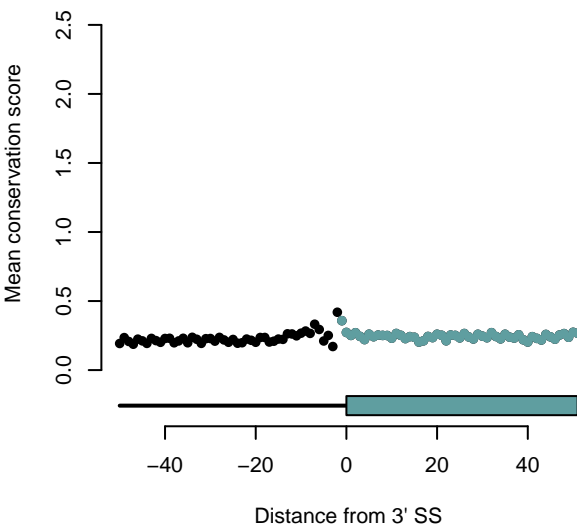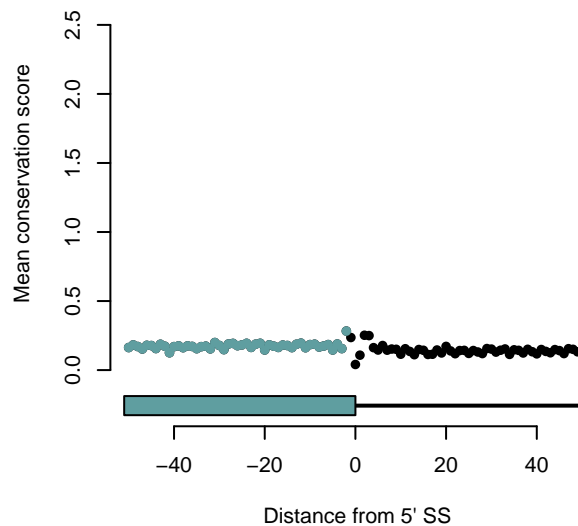

Supplement: Figure S3 — Unannotated splice sites identified in a different population of LCLs are unconserved. This figure is identical to Figure 3 in the main paper, except that it uses splice sites identified in the European-ancestry LCLs. (0.05 MB PDF) [file pgen.1001236.s003.pdf]

## Annotated splice sites

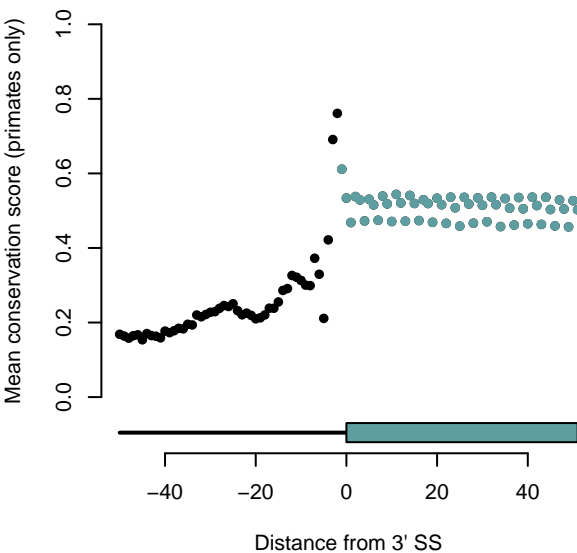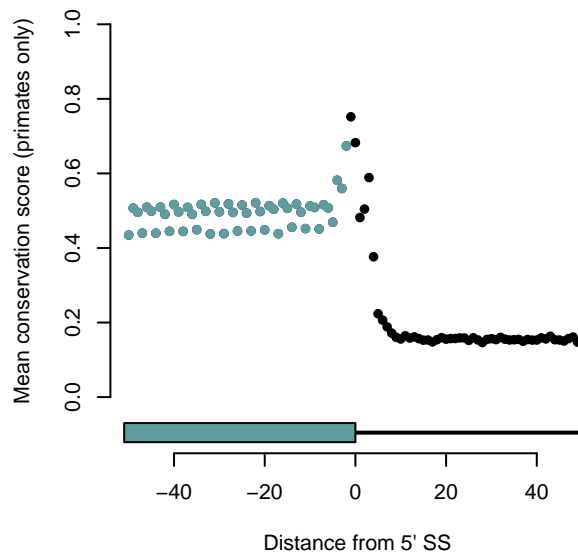

## Unannotated splice sites

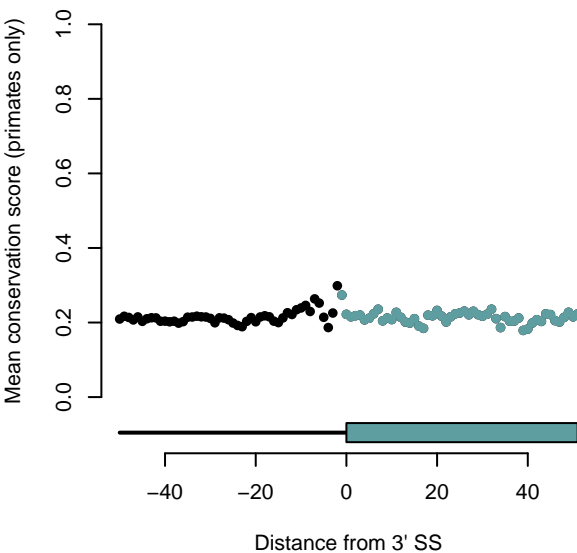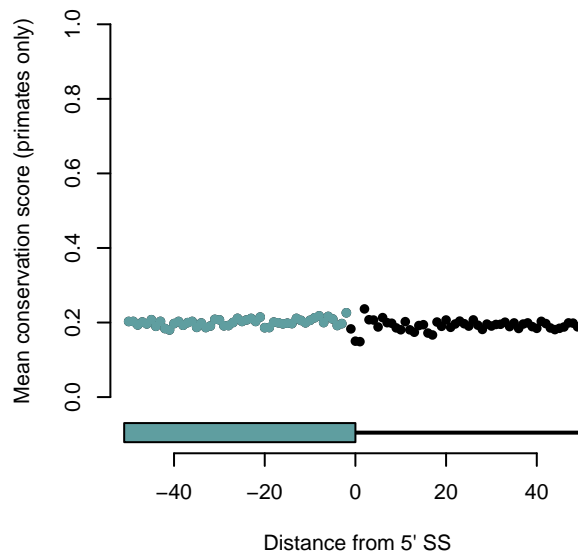

Supplement: Figure S4 — Unannotated splice sites are not conserved across primate evolution. This figure is identical to Figure 3 in the main paper, except we used phyloP score calculated in the primate phylogeny, rather than the mammalian phylogeny. (0.05 MB PDF) [file pgen.1001236.s004.pdf]

## Annotated splice sites

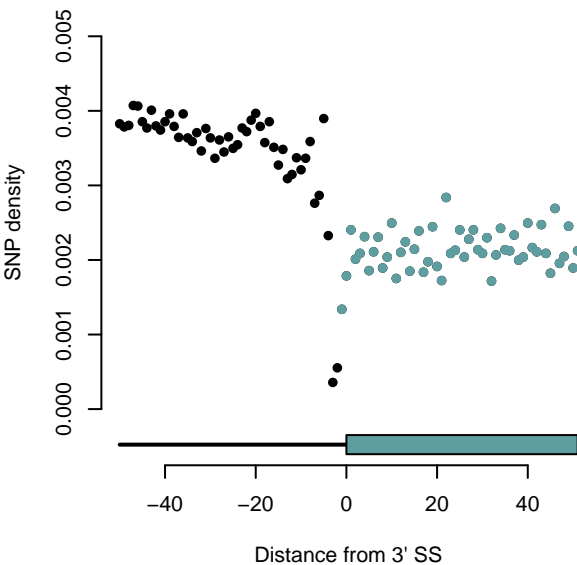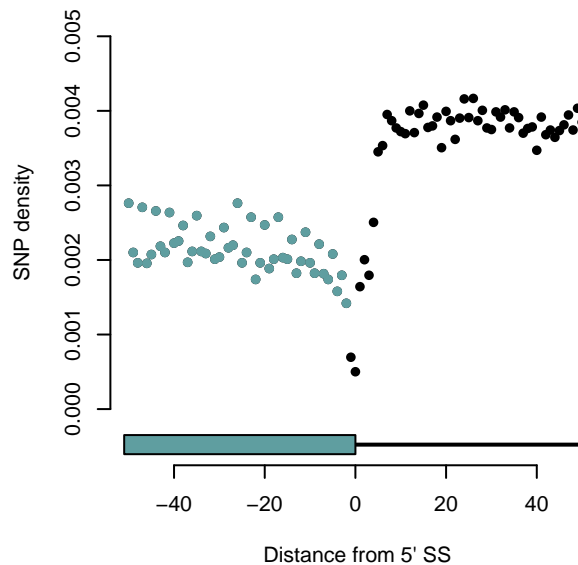

## Unannotated splice sites

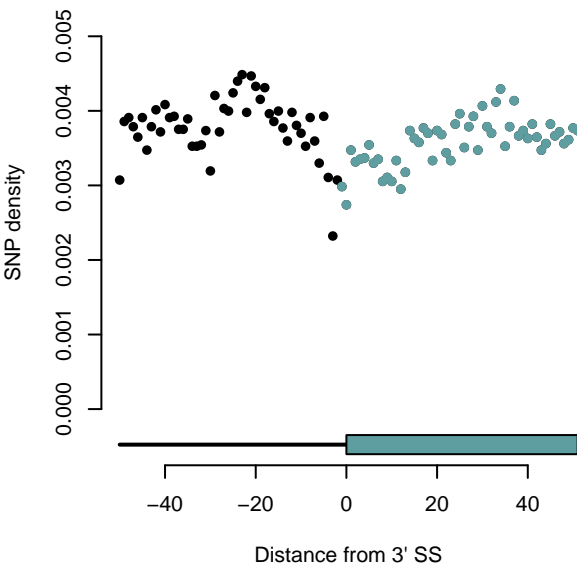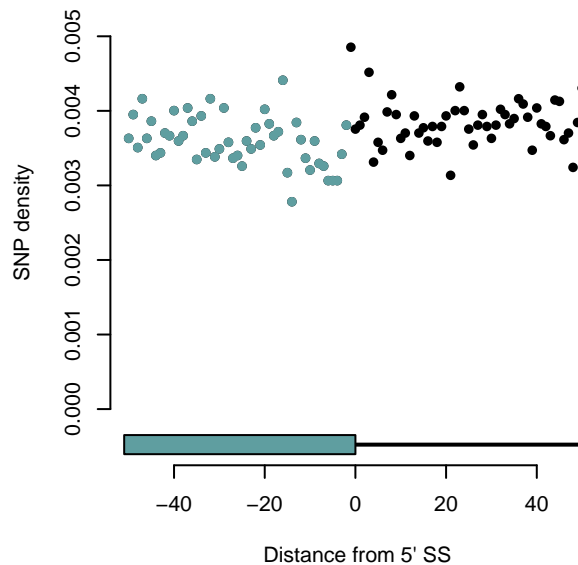

Supplement: Figure S5 — Unannotated splice sites show no reduction of polymorphism levels in humans. We used data from the 1,000 Genomes Project to calculate the SNP density in and around splice sites. For each splice site (annotated or unannotated), we used the SNP calls in the Yoruban population to evaluate whether there is any polymorphism at each position at a distance from each site. Plotted is that fraction of sites that have a polymorphism in the population at each position away from the splice site. Annotated and unannotated splice sites are plotted separately. There is a clear reduction of polymorphism directly intronic of the annotated splice sites, but no such reduction intronic of the unannotated splice sites. (0.05 MB PDF) [file pgen.1001236.s005.pdf]

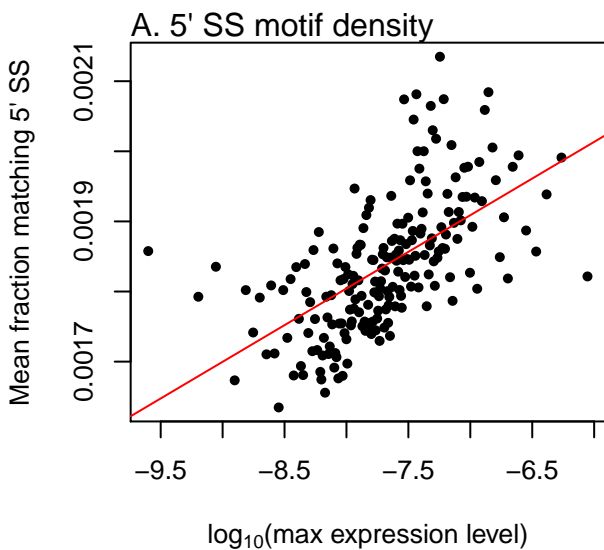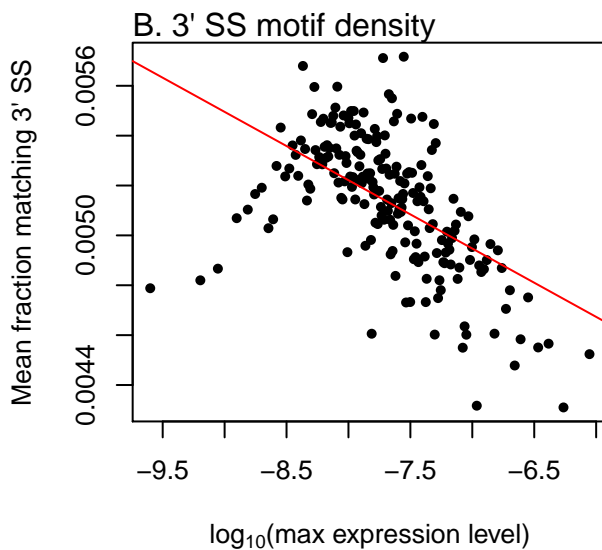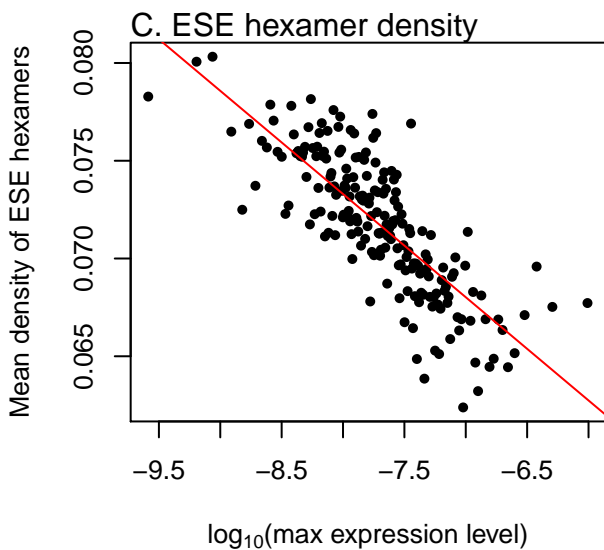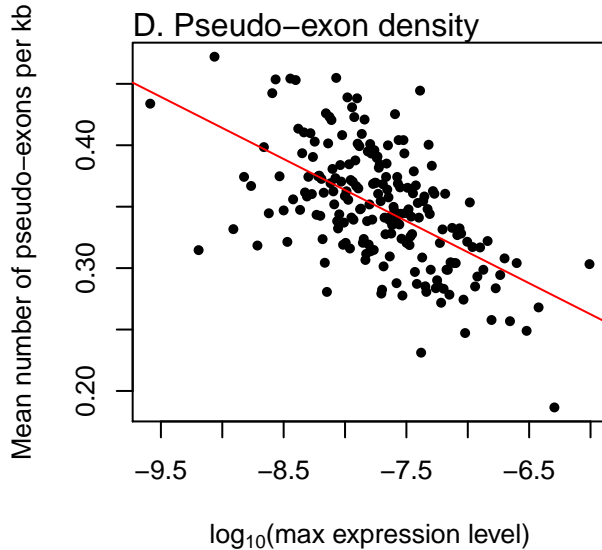

Supplement: Figure S7 — Sequence analysis of introns. A. 5′ splice site motif density in introns correlates with gene expression level. We calculated the density of matches to the 5′ splice site motif (see Text S1) in each intron, then grouped all introns into 200 bins based on the expression level of the gene in which each falls. Plotted is the mean density of matches to the motif against the mean expression level in each bin. B. 3′ splice site motif density in introns correlates with gene expression level. The same plot as in A., except the y-axis is the density of matches 3′ splice site motif. C. ESE hexamer density in introns correlates with gene expression level. As in A., except the y-axis is the density of matches to the putative ESEs identified by Fairbrother et al. (2002). D. Pseudo-exon density in introns correlates with gene expression level. As in A, except the y-axis is the density of pseudo-exons (see ). (0.06 MB PDF) [file pgen.1001236.s007.pdf]
