# Supplementary material for: Noisy Splicing Drives mRNA Isoform Diversity in Human Cells
Source: PLoS Genet. 2010 Dec 9;6(12):e1001236. doi: 10.1371/journal.pgen.1001236 (PMC3000347; doi:10.1371/journal.pgen.1001236)
Supplement: Figure S6 — The low splicing error rate of highly-expressed genes is largely due to their small intron sizes. A. Splicing error rate correlates with intron length. This is simply a re-plotted version of Figure 3 in the main text. All highly-conserved introns were grouped into 100 bins based on length; plotted is the mean splicing error rate in the bin against the mean intron length in the bin. B. Correction for gene expression level does not influence the correlation between splicing error rate and intron length. We corrected the observed splicing error rates for gene expression level (see Text S1), and performed the same analysis as in A. C. Splicing error rate correlates with gene expression level. All highly-conserved introns were grouped into 100 bins based on the gene expression level of the gene in which they fall; plotted is the mean splicing error rate in the bin against the mean expression level in the bin. D. Correction for intron length removes the correlation between splicing error rate and expression level. We corrected the observed splicing error rates for intron length (see Text S1), and performed the same analysis as in C. (0.04 MB PDF) [file pgen.1001236.s006.pdf]

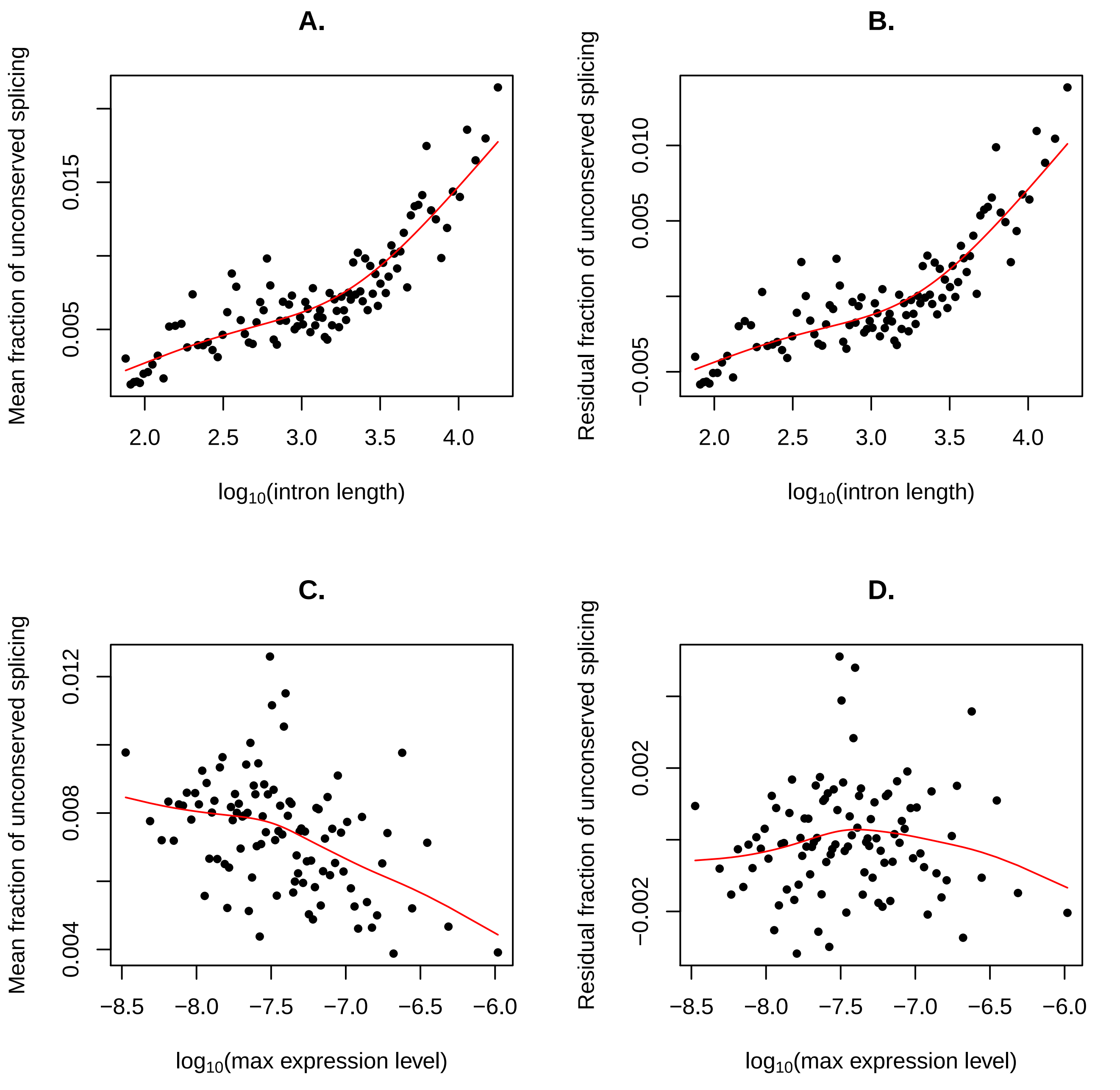
A.
Mean fraction of unconserved splicing
0.005
0.015
2.0
2.5
3.0
3.5
4.0
log10(intron length)
B.
Residual fraction of unconserved splicing
−0.005
0.005
0.010
2.0
2.5
3.0
3.5
4.0
log10(intron length)
C.
Mean fraction of unconserved splicing
0.004
0.008
0.012
−8.5
−8.0
−7.5
−7.0
−6.5
−6.0
log10(max expression level)
D.
Residual fraction of unconserved splicing
−0.002
0.002
−8.5
−8.0
−7.5
−7.0
−6.5
−6.0
log10(max expression level)
