# Supplementary material for: Noisy Splicing Drives mRNA Isoform Diversity in Human Cells
Source: PLoS Genet. 2010 Dec 9;6(12):e1001236. doi: 10.1371/journal.pgen.1001236 (PMC3000347; doi:10.1371/journal.pgen.1001236)
Supplement: Figure S8 — Rarely-used but annotated splice sites are highly conserved. As for Figure 3 in the main text, we identified all the splice junctions where one end is annotated and the other is not. We then limited ourselves to splice sites covered by exactly one read in our data (there are approximately 10,000 such annotated splice sites and 20,000 unannotated splice sites of each type), and performed the same analysis of conservation in the main text. The top panel shows the results for the annotated splice sites, and the bottom panel the results for the unannotated splice sites. The marked difference between the two classes remains. (0.05 MB PDF) [file pgen.1001236.s008.pdf]

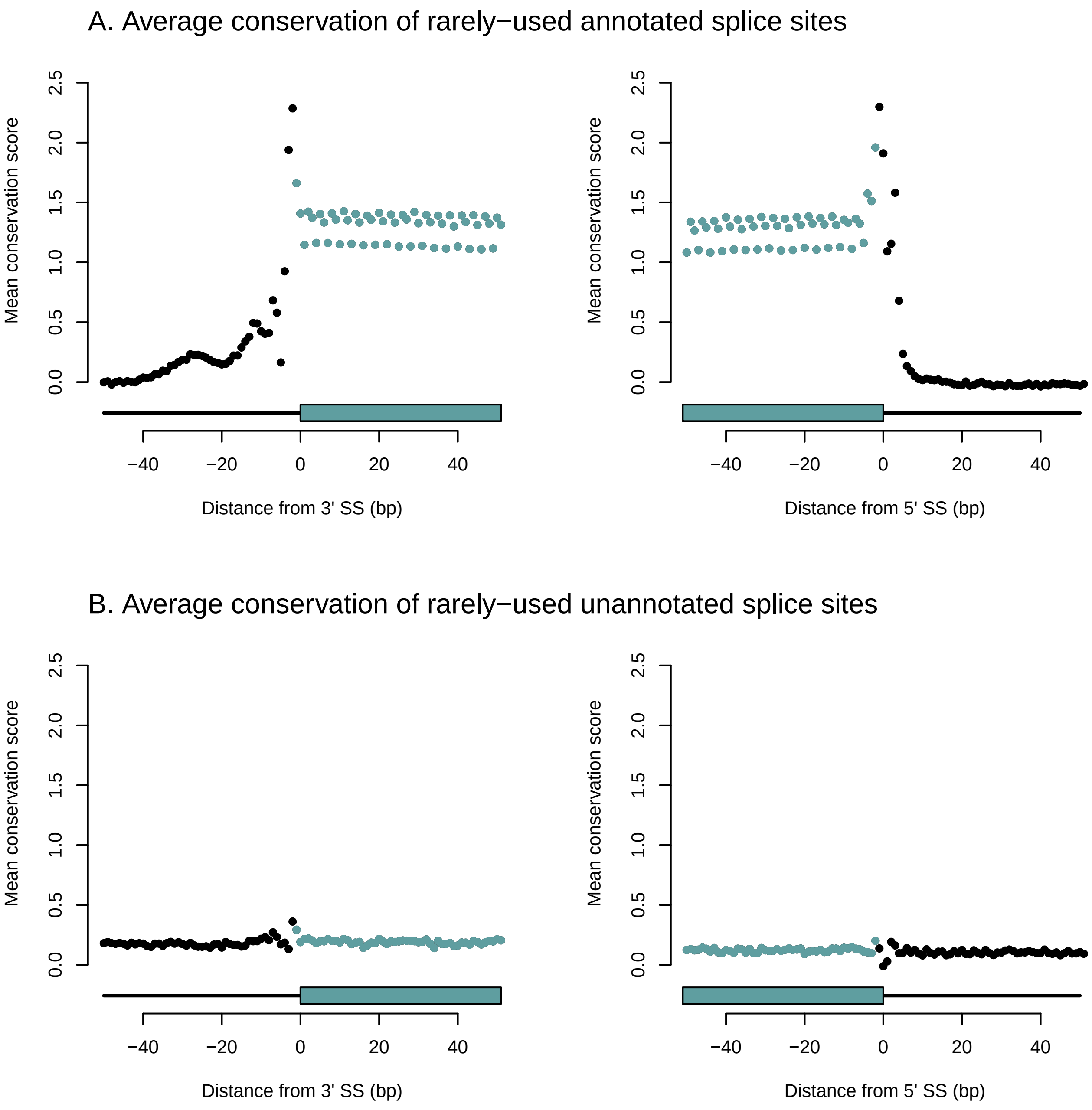
A. Average conservation of rarely−used annotated splice sites
Mean conservation score
0.0
0.5
1.0
1.5
2.0
2.5
−40
−20
0
20
40
Distance from 3' SS (bp)
Distance from 5' SS (bp)
B. Average conservation of rarely−used unannotated splice sites
Mean conservation score
Distance from 3' SS (bp)
Distance from 5' SS (bp)
