# Supplementary material for: Noisy Splicing Drives mRNA Isoform Diversity in Human Cells
Source: PLoS Genet. 2010 Dec 9;6(12):e1001236. doi: 10.1371/journal.pgen.1001236 (PMC3000347; doi:10.1371/journal.pgen.1001236)
Supplement: Text S1 — Supplementary text. (0.09 MB PDF) [file pgen.1001236.s009.pdf]

Supplementary Text for: Noisy splicing drives mRNA isoform diversity in human cells

Joseph K. Pickrell[1,†], Athma A. Pai[1,†], Yoav Gilad[1,†], Jonathan K. Pritchard[1,2,†]

[1] Department of Human Genetics, [2] Howard Hughes Medical Institute, The University of Chicago.

[†] Corresponding authors: pickrell@uchicago.edu, athma@uchicago.edu, gilad@uchicago.edu, pritch@uchicago.edu

September 14, 2010

Estimation of the amount of individual-specific splicing. We asked whether the extent of unannotated splicing we observed might be partially attributable to splicing events limited to a fraction of the individuals in our sample, due to sequence polymorphisms which influence splicing. Though we have little power to detect such polymorphisms in these samples (Pickrell et al., 2010), if sequence polymorphisms with strong influences on splicing are prevalent there should be other patterns in the data which indicate this. First, a significant fraction of the splice junctions we see should be seen in only a single individual (under the assumption that many polymorphisms in a sample are individual-specific). In fact, only 47 junctions covered by more than three sequencing reads are limited to a single individual (this is 0.03% of all the splice junctions with more than three reads). Manual inspection of these junctions showed that several are due to genes expressed in only a single individual (rather than individual-specific splicing), and we could identify only one putative case of a rare sequence polymorphism causing the generation of an alternative isoform.

The above confirms that the overwhelming majority of isoforms present at any appreciable frequency are not individual-specific. We then considered the possibility that the very low-abundance isoforms we observed might be enriched for individual-specific variants. This might be the case if, for example, polymorphisms often have a very small influence on splicing. To test this, we used the set of splice junctions covered by exactly two sequencing reads (there are over 32,000 of these). If the rate at which each splice form is generated is constant across individuals, the two reads from each junction should not be more likely to come from the same individual than two random reads. In fact, in about 6% of the cases, both reads come from the same individual, while we would expect 1.5% of them to come from the same individual by chance. This would seem to suggest that about 4.5% of these splice junctions do not fit the model of uniform sampling. However, on examination of the splice junctions with two reads from the same individual, we noticed that the two reads were often identical (55% of the 2074 splice junctions with exactly two reads, both from the same individual); this was not the case when the two reads were from different individuals. This suggested that PCR biases during library preparation were confounding this analysis (see, for example, Quail et al. (2008) and Kozarewa et al. (2009)); we estimate a duplication rate of 1.2% (see below). If we remove putative duplicate reads from the analysis, this now suggests that about 1.5% of splice junctions with two reads do not fit the model of uniform sampling. We regard this as an upper bound for the amount of individual-specific splicing, as other unknown library preparation biases may contribute to this estimate.

Estimation of the duplication rate of RNA-Seq reads. As described above, we noticed that sometimes the exact same read was present multiple times in the same individual. Since for most individuals only a single sequencing library was prepared, these duplicate reads are likely PCR artifacts (Kozarewa et al., 2009; Quail et al., 2008). We wanted to estimate the fraction of sequencing reads that might be due to such effects. We identified all splice junctions present in only a single individual and for which all the sequencing reads covering the junctions are identical. If there are N such splice junctions, and we assume that all of the reads seen more than once are due to duplications, then the expected number of duplicated reads for every true read is:

$$E[dup] = 1 - \sum_i i \frac{n_i}{N} \quad (1)$$

where n_i is the number of splice junctions covered by i identical reads. In our data, this value is 0.012, suggesting that about 1.2% of reads are PCR duplicates.

The impact of intron length and gene expression level on splicing error rates. In the main text, we show that larger introns have increased splicing error rates (Figure S6A; $\rho = 0.83$, $P < 2 \times 10^{-16}$). We also saw that highly expressed genes also tend to have lower splicing error rates (Figure S6C; $\rho = -0.43$, $P = 7 \times 10^{-6}$). Since these two correlations are somewhat confounded (Castillo-Davis et al., 2002), we considered whether the effects of one remains after correction for the other. First, we calculated, as in the main text, the splicing error rate of each intron. Let this be e_i, where i indexes the intron. Then, we split all introns into 100 bins based on their length, and calculated the mean splicing error rate for introns in each bin, as well as the mean intron length. We then fit a spline to these points (Figure S6A). This was done using the smooth.spline function in R, with a smoothing parameter of 1. For each intron, then, we can calculate the expected rate of splicing error given the intron length. Let this be $\hat{e}_i$. We can then calculate the residual, r_i from this fit:

$$r_i = e_i - \hat{e}_i \quad (2)$$

We then split introns into 100 bins based on expression level, and calculated the mean of the r_i in each bin, as well as the mean of the expression level in each bin. After the correction, there is no correlation between expression level and splicing error rate (Figure S6D; $\rho = 0.04$, $P = 0.66$). When we do the reverse correction, we find no reduction in the correlation between intron length and splicing error rate (Figure S6B; $\rho = 0.83$, $P < 2 \times 10^{-16}$).

Sequence analysis of introns in highly expressed genes. We also considered the possibility that the sequences of introns in highly expressed genes have evolved to contain fewer sequences that could spuriously be recognized as exons by the cell. For each intron identified in the LCLs, we calculated the density of motifs matching the consensus 5' splice site, the consensus 3' splice site (to judge this, we used a position weight matrix derived from Ensembl; see below), and the density of hexamers matching putative exonic splicing enhancers (ESEs) (Fairbrother et al., 2002). We find that there is a negative correlation between the density of putative ESEs and the 3' splice site motif in introns and the expression level of a gene, as well as a modest positive correlation between the density of the 5' splice site motif in introns and the expression level of a gene (Figure S7). Combining these factors, we counted the density of "pseudo"-exons (matches to the 3' and 5' splice sites located 100-300 bases apart, with an ESE hexamer density of at least 0.1 between them) in each intron. Introns in highly expressed genes are depleted for pseudo-exons relative to introns in lowly-expressed genes (Figure S7). This would seem to indicate that the sequences of introns in highly expressed genes have evolved to reduce the number of potential spurious exons.

However, two additional predictions of this model are not borne out. First, we would expect that the exons of highly expressed genes should have higher ESE density than exons in lowly expressed genes; in fact, the opposite is true (not shown). Second, to control for differences in sequence composition between introns at different expression levels, we compared the density of pseudo-exons in introns with that in control introns generated by permuting the sequences of each intron. We expected that highly expressed genes should be more depleted of pseduo-exons than lowly expressed genes in this analysis; in fact, we saw no such relationship (not shown). We conclude that, while there is slightly suggestive evidence that that selection against splicing errors has influenced the sequences of introns in highly expressed genes, this is most likely an artifact driven by difference in sequence composition of genomic regions containing these genes.

Position weight matrices for the splice sites. To build position weight matrices for the consensus splice sites, we extracted three bases exonic and six bases intronic of each 5' splice site, and 20 bases intronic and two bases exonic of every 3' splice site annotated in Ensembl. We then estimated the fraction of each of the four bases at each position, and used this as our PWM. To judge how well a given sequence matches each PWN, we simply used a log-likelihood ratio from multiplying together the marginal probabilities at each base:

$$l(S) = \sum_i \log(P(S_i)/0.25), \tag{3}$$

where S is the sequence, $P(S_i)$ is the probability from the PWM of seeing the base at position i in the sequence at position i in the PWM, and 0.25 is the background probability (which we set to be uniformly distributed on all four bases). For the above analysis, we used a log-likelihood ratio threshold of 2 as a "match" to the PWM.

Table 1: **Summary of new data produced**. For each lane, we give the sequencing center where the lane was sequenced, the cell line ID, the total number of reads and the number of reads that mapped (uniquely or not) to the genome (excluding splice junctions). All data are available at http://eqtl.uchicago.edu.

Center	Ind	Total	Mapped
yale	GM18859	10007505	8719400
yale	GM19092	11123460	9806511
yale	GM19102	8971294	7995293
yale	GM19141	9626823	8639032
yale	GM19206	11010264	9588663
yale	GM19207	11044860	9879829
argonne	GM18859	12279759	10333351
argonne	GM19092	12762642	10664340
argonne	GM19102	10151922	8577579
argonne	GM19141	9778473	8407019
argonne	GM19206	10756275	8700526
argonne	GM19207	10454167	8828939

References

Castillo-Davis, C. I., Mekhedov, S. L., Hartl, D. L., Koonin, E. V., and Kondrashov, F. A., 2002. Selection for short introns in highly expressed genes. *Nat Genet*, **31**(4):415–8.

Fairbrother, W. G., Yeh, R.-F., Sharp, P. A., and Burge, C. B., 2002. Predictive identification of exonic splicing enhancers in human genes. *Science*, **297**(5583):1007–13.

Kozarewa, I., Ning, Z., Quail, M. A., Sanders, M. J., Berriman, M., and Turner, D. J., 2009. Amplification-free Illumina sequencing-library preparation facilitates improved mapping and assembly of (G+C)-biased genomes. *Nat Methods*, **6**(4):291–5.

Pickrell, J. K., Marioni, J. C., Pai, A. A., Degner, J. F., Engelhardt, B. E., Nkadori, E., Veyrieras, J.-B., Stephens, M., Gilad, Y., and Pritchard, J. K., *et al.*, 2010. Understanding mechanisms underlying human gene expression variation with RNA sequencing. *Nature*, **464**(7289):768–72.

Quail, M. A., Kozarewa, I., Smith, F., Scally, A., Stephens, P. J., Durbin, R., Swerdlow, H., and Turner, D. J., 2008. A large genome center's improvements to the Illumina sequencing system. *Nat Methods*, **5**(12):1005–10.
